# Spatial Variability of Antarctic Surface Snow Bacterial Communities

**DOI:** 10.3389/fmicb.2019.00461

**Published:** 2019-03-26

**Authors:** Lucie A. Malard, Marie Šabacká, Iordanis Magiopoulos, Matt Mowlem, Andy Hodson, Martyn Tranter, Martin J. Siegert, David A. Pearce

**Affiliations:** ^1^Department of Applied Sciences, Faculty of Health and Life Sciences, Northumbria University at Newcastle, Newcastle upon Tyne, United Kingdom; ^2^Centre for Polar Ecology, University of South Bohemia in České Budějovice, České Budějovice, Czechia; ^3^Institute of Oceanography, Hellenic Centre for Marine Research, Heraklion, Greece; ^4^Ocean Technology and Engineering Group, National Oceanography Centre Southampton, Southampton, United Kingdom; ^5^Arctic Geology, University Centre in Svalbard, Longyearbyen, Norway; ^6^Department of Environmental Sciences, Western Norway University of Applied Sciences, Bergen, Norway; ^7^Bristol Glaciology Centre, University of Bristol, Bristol, United Kingdom; ^8^Grantham Institute, Department of Earth Science and Engineering, Imperial College London, London, United Kingdom; ^9^British Antarctic Survey, Natural Environment Research Council, Cambridge, United Kingdom

**Keywords:** Antarctic, snow, biogeography, Ellsworth Lake, microbial diversity, relic DNA

## Abstract

It was once a long-held view that the Antarctic was a pristine environment with low biomass, low biodiversity and low rates of microbial activity. However, as the intensity of scientific investigation has increased, so these views have started to change. In particular, the role and impact of human activity toward indigenous microbial communities has started to come under more intense scrutiny. During the Subglacial Lake Ellsworth exploration campaign in December 2012, a microbiological survey was conducted to determine the extent and likelihood of exogenous input into the subglacial lake system during the hot-water drilling process. Snow was collected from the surface to represent that used for melt water production for hot-water drilling. The results of this study showed that snow used to provide melt water differed in its microbiological composition from that of the surrounding area and raised the question of how the biogeography of snow-borne microorganisms might influence the potential outcome of scientific analyses. In this study, we investigated the biogeography of microorganisms in snow around a series of Antarctic logistic hubs, where human activity was clearly apparent, and from which scientific investigations have been undertaken. A change in microbial community structure with geographical location was apparent and, notably, a decrease in alpha diversity at more remote southern latitudes. Soil-related microorganisms dominated microbial assemblages suggesting terrestrial input, most likely from long-range aeolian transport into continental Antarctica. We also observed that relic DNA was not a major issue when assessing snow samples. Overall, our observations might have profound implications for future scientific activities in Antarctica, such as the need to establish “no-go” protected areas, the need for better characterization of field sites and improved protocols for sterilization and verification of ice drilling equipment.

## Introduction

The cryosphere covers about 20% of Earth’s surface (Boetius et al., 2015) and seasonally snow-covered areas cover up to 35% of the terrestrial surface (Miteva, 2008). Snow represents a climatically sensitive transient ecosystem, linking the atmosphere to the soil or ice below (Kuhn, 2001); it is physically, chemically and biologically dynamic, has a strong impact on the hydrological cycle, and interacts with a large number of other ecosystems worldwide (Vavrus, 2007; Callaghan et al., 2011). Pristine snow is primarily seeded from the atmosphere and unique microbial communities thus develop within the snow over time (Harding et al., 2011; Larose et al., 2013), which then actively participate in nutrient cycling (Jones, 1999). Within the snow, microorganisms encounter very low temperatures, variable UV radiation (at the surface), limited water availability and oligotrophic nutrient conditions (Larose et al., 2013). Yet, microorganisms living in such habitats have been identified worldwide, including within glacial ice (Christner et al., 2000), cryoconite holes (Margesin et al., 2002), sea ice (Staley and Gosink, 1999) and snow (Carpenter et al., 2000). Antarctic snow is an important habitat as over 99% of the continent is permanently ice covered (Fretwell et al., 2013) and microbial life dominates any other form of terrestrial life there (Vincent, 1988).

Studies of snow-borne microorganisms have mainly focused on Arctic regions, with fewer studies on Alpine and Antarctic snow communities. Arctic snow has been shown to harbor primarily Proteobacteria (Alpha- and Beta-), along with Bacteroidetes, Firmicutes, and Cyanobacteria (Harding et al., 2011; Hell et al., 2013; Larose et al., 2013; Møller et al., 2013; Maccario et al., 2014). The two large high throughput sequencing studies on Alpine snow microbes show a dominance of Alphaproteobacteria and Betaproteobacteria, with variable relative abundances of Firmicutes, Bacteroidetes, Acidobacteria, Actinobacteria and Cyanobacteria (Wunderlin et al., 2016; Azzoni et al., 2018). While more recent studies have investigated the physico-chemical properties of Antarctic snow (Hodson et al., 2017), patterns of accumulation and ablation (Eveland et al., 2013) and optical properties (France et al., 2011), only a limited number have investigated microbial diversity and distribution, and these report high abundances of Proteobacteria (Alpha-, Beta-) and variable levels of Firmicutes, Bacteroidetes and Cyanobacteria (Michaud et al., 2014; Antony et al., 2016; Lopatina et al., 2016). These studies are restricted to a limited number of specific locations leaving the vast majority of the continent unexplored. Hence, broad scale biogeographic patterns of Antarctic snow microorganisms are unknown. In addition, knowledge of colonization pathways, and whether Antarctic ecosystems are seeded primarily by local dispersal processes or long-range transport, is still open to question (Pearce et al., 2016).

There have been a number of Antarctic subglacial lake access projects in recent years such as those aiming to explore Lake Vostok in East Antarctica (Bulat, 2016), Lake Whillans at the edge of the West Antarctic Ice Sheet (Tulaczyk et al., 2014; Siegert et al., 2016), Lake Ellsworth in the center of West Antarctica (Siegert et al., 2014) and the recent ‘subglacial Antarctic lakes scientific access’ (SALSA) project focused on Lake Mercer and Lake Engelhardt on the Whillans Ice Plain. While these programs follow strict protocols to avoid microbial contamination of the subglacial ecosystems they intend to access (Priscu et al., 2013; Makinson et al., 2016), they have exposed an issue that little attention is being given to indigenous microbial communities at the snow surface and what impact they might potentially have on the results obtained. For example, it is not yet known whether the snow surface communities enter the subglacial system directly via migration through the ice or indeed anthropogenically via ineffective sterilization of hot water drill fluid. Elsewhere, studies have shown the presence of human-associated bacteria in the vicinity of research stations (Hughes and Thompson, 2004) and field camps (Sjoling and Cowan, 2000), suggesting a potential impact of human activity, including in deep-field locations (Cowan et al., 2011).

Here, we investigate the spatial distribution of microbial communities in Antarctic surface snow in areas close to logistic hubs and areas of strong scientific research interest, from Signy Island to the Ellsworth Mountains. We also characterized the proportion of the bacterial community that was potentially viable using a propidium monoazide (PMA) assay, thus allowing us to assess the impact of relic DNA on Antarctic snow diversity studies. From these analyses, we identified patterns of microbial distribution and spatial variability within the Antarctic surface snowpack, and assessed bacterial viability. Our investigation underlines the importance of a full understanding of the microbiology of the field site prior to invasive field operations.

## Materials and Methods

### Sample Collection

**FIGURE 1:**
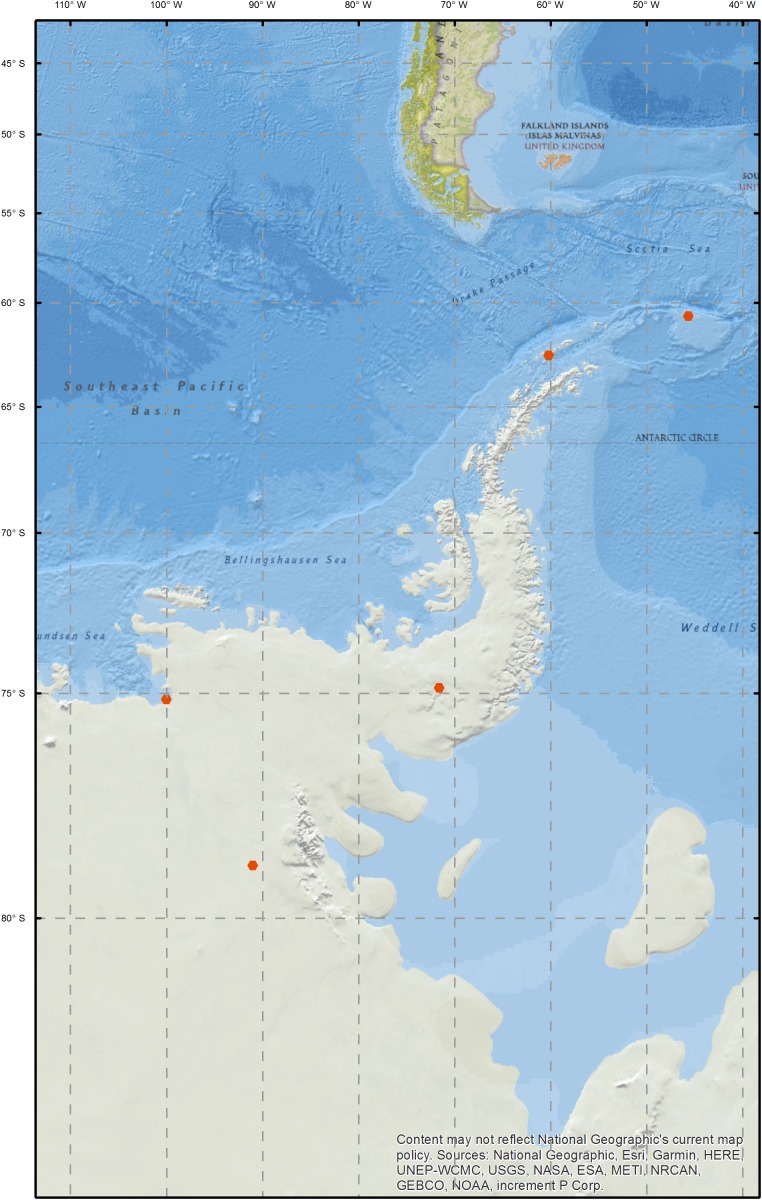
Map of sampling sites.

Snow samples were collected between the South Orkney Islands and the Ellsworth Mountains between December 2012 and January 2014 (Figure 1 and Supplementary Table 1). On Signy Island, two sites were sampled; Gourlay Snowfield and Tuva Glacier. Signy Island is an important bird area and hosts a research station, maintained by the British Antarctic Survey (BAS). While the sampled areas are not directly impacted by the research station, there is human presence on the island and ongoing research activity in the vicinity. Livingston Island samples are described in the study by Hodson et al. (2017), who investigated the relationship between microbial communities and optical and physico-chemical properties of Antarctic snow. Livingston Island hosts five scientific bases, and occasional field camps. SkyBlu samples came from the vicinity of the BAS blue ice runway (a logistics hub for deep field operations). Pine Island Bay area is of research interest as the Pine Island Glacier and Thwaites Glacier are fast retreating and their collapse could lead to major sea level rise (Rignot et al., 2014). Ellsworth off-site samples were collected approximately one kilometer upwind of the Lake Ellsworth deep-field camp (Pearce D.A. et al., 2016). Camp samples were collected around the kitchen, generator and drilling areas. Snow was collected from the surface to represent collection during melt water production for hot-water drilling. At each sampling location, a 1 m snow pit was excavated and the top 30 cm of snow was sampled using an ethanol sterilized shovel and Whirl-Pak bags (Nasco, WI, United States). Samples were transported to the field laboratory, where they were melted and passed through 0.2 μm Sterivex filters (Merck, Darmstadt, Germany), before freezing at −20°C for transport and processing in the United Kingdom.

### PMA Treatment and DNA Extractions

SkyBlu, Pine Island Bay and Ellsworth snow samples were processed in two ways; PMA-treated and non-PMA-treated in order to differentiate the potentially viable microbial community from relic DNA. Each 0.2 μm filter was cut in half using sterile and DNAase/ethanol treated razors. For each sample, one-half was processed with PMA as per Nocker and Camper (2009) and Fittipaldi et al. (2012), using a 20 mM stock solution of PMA (Biotium, Hayward, CA, United States) in a 20% (v/v) aqueous solution of dimethyl sulfoxide (DMSO). Filters for PMA treatment were placed in a 6-well plate and a PMA solution at a final concentration of 100 μM was added. Cross-linking was initiated by 10 min incubation on ice, in the dark with occasional mixing, followed by 5 min of light exposure using a 650 W halogen lamp (FLASH 2000 L, DTS, Italy), at a 20 cm distance. The process was carried out in a laminar flow hood to avoid contamination of the samples. Non-PMA-treated samples were incubated in a 20% (v/v) aqueous solution of DMSO, and treated following the same protocol as PMA-treated samples. All samples were washed twice with sterile phosphate buffered saline (PBS) and all samples were then used for DNA extraction.

DNA from snow samples was extracted using the PowerWater kit from MoBio (Qiagen, Carlsbad, CA, United States). Each sample was PCR amplified using the primers 341F and 785R covering the V3-V4 regions of the 16S rRNA gene (Klindworth et al., 2013), under the following conditions: initial denaturation at 95°C for 5 min then 25 cycles of 40 s denaturation at 95°C; primer annealing at 55°C for 2 min; and elongation at 72°C for 1 min then a final elongation at 72°C for 7 min (Hodson et al., 2017). DNA extraction kit controls were included alongside the snow derived DNA and sequenced. PCR amplicons were cleaned, normalized, quantified and supplemented with 5% PhiX before being loaded on Illumina MiSeq, as per the Illumina standard operating protocol (Kozich et al., 2013).

### Illumina Sequencing and Data Processing

Forward and reverse reads longer than 420 bp were merged using FLASH (fast length adjustment of short reads) (Magoč and Salzberg, 2011) for a total of 4.2 million reads. Vsearch (Rognes et al., 2016) was used for downstream analyses. Quality filtering by expected errors was run to keep only sequences with high Phred scores. Dereplication was used to identify unique sequences. Chimeras were removed using a two-step chimera detection method; first by aligning against ChimeraSlayer Gold database provided with SILVA (Pruesse et al., 2007), and secondly by using the *de novo* detection module in Vsearch. *De novo* operational taxonomic unit (OTU) calling was carried out at a 97% similarity level to generate operational taxonomical units (OTUs), and aligned using the Python Nearest Alignment Space Termination (PyNAST) (Caporaso et al., 2009) tool with a relaxed neighbor-joining tree built using FastTree (Price et al., 2010). The taxonomy was determined using the Classification Resources for Environmental Sequence Tags (CREST) (Lanzén et al., 2012) classifier with a confidence threshold of 0.80 against SILVA release 128 as a reference database. A total of 42 DNA samples, equal to 3 million reads (63,202 ± 60,525 reads/sample), were assigned against 6,793 OTUs using the USEARCH (Edgar, 2010) algorithm for clustering with a threshold of 97 % similarity. Datasets are deposited on the European Nucleotide Archive under the accession numbers SMAN06368443 to SAMN06368447 and PRJEB29215.

### Statistical Analysis

All statistical analyses were performed with a combination of Qiime1 V 1.90 (Caporaso et al., 2010a) and R environment (R Core Team, 2013) using phyloseq (McMurdie and Holmes, 2013), vegan (Dixon, 2003) and indicspecies (Cáceres and Legendre, 2009) packages. We calculated the alpha diversity using matrices of richness (number of observed OTUs) and diversity (Shannon and Simpson indices) based on OTU tables rarefied to the lower number of reads. We tested differences in alpha diversity using ANOVA and Tukey honestly significant differences (HSD) test to define which location exhibited significant differences. Beta diversity using Bray-Curtis distance was then calculated by normalizing the OTU table using cumulative-sum scaling (CSS), in which raw counts are divided by the cumulative sum (Paulson et al., 2013). The dissimilarity matrix was plotted using principal coordinates analysis (PCoA). ANOSIM from vegan was used to analyze the similarities based on a Bray-Curtis dissimilarity matrix across locations with free permutations. Indicator species were determined by the Dufrene-Legendre indicator species analysis method (Cáceres and Legendre, 2009) to identify OTUs that were specifically associated with the different geographic locations.

## Results

### The Surface Snow

**FIGURE 2:**
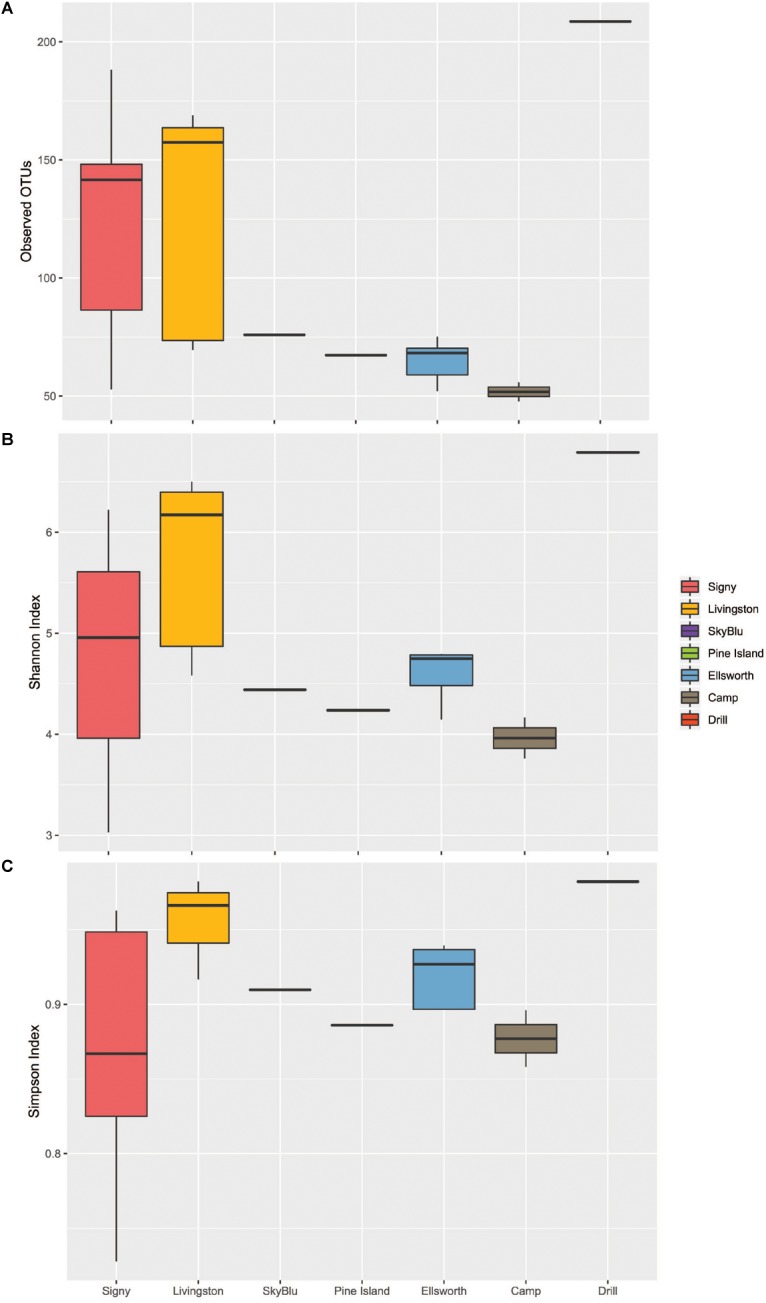
Boxplots of Alpha diversity measures. Ellsworth indicates Ellsworth pristine samples. Camp samples include those from the kitchen and generators of the deep field camp at Lake Ellsworth, while the drill sample represents that collected close to the hot-water drilling hole. **(A)** Observed OTUs by Location. **(B)** Shannon Index by Location. **(C)** Simpson Index by Location.

We first investigated the bacterial communities excluding the Ellsworth field camp samples from the analysis as these were specifically chosen to maximize the chance of detecting some human impact. The alpha diversity analysis identified geographic differences in the number of observed OTUs (ANOVA *F*_6,25_ = 4.49, *p* = 0.0032) and Tukey’s HSD test suggested a latitudinal gradient with significant differences between Signy Island and the Ellsworth samples (Tukey HSD, *p* = 0.016). The Shannon and Simpson indices showed that samples from Livingston were more diverse and even that at any other location. Overall, Pine Island Bay, SkyBlu and Ellsworth harbored fewer OTUs than Livingston and Signy Island samples (Figure 2A–C).

**FIGURE 3:**
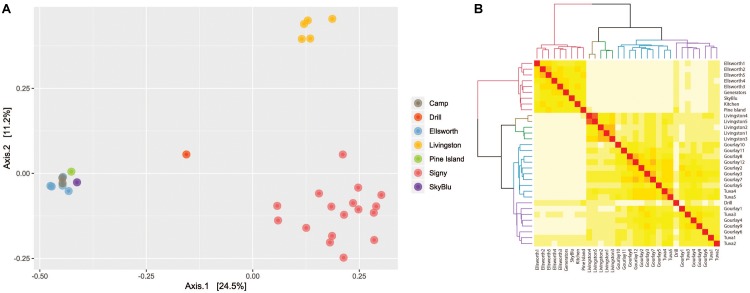
**(A)** Principal component analysis based on Bray-Curtis distance illustrating the differences between bacterial communities by geographic location. **(B)** Heatmap of Bray-Curtis dissimilarity matrix with dendrogram of samples illustrating geographic clustering.

Principal component analysis of bacterial communities identified three clusters (Figure 3A), corresponding to three locations: Livingston Island, Signy Island and continental Antarctica (encompassing the Lake Ellsworth area, Pine Island Bay and SkyBlu). A heatmap and dendrogram (Figure 3B) further illustrated the grouping into the three distinct clusters by geographic locations; each Antarctic island and continental Antarctica. Not only were each of the clusters different, samples within clusters differed in relative abundance and composition, as highlighted in the PCoA (Figure 3A) and heatmap (Figure 3B) (ANOSIM *R* = 0.782, *p* < 0.001).

**FIGURE 4:**
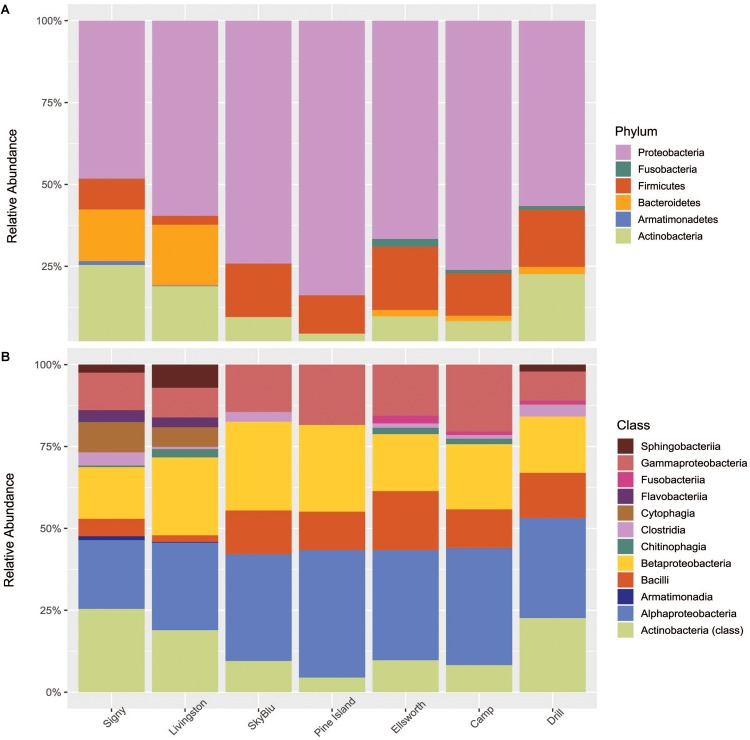
Stacked bar chart of the dominant 0.1% bacterial diversity by sampling site **(A)** at the phylum level, and **(B)** at the class level.

Of 6,793 taxa identified, only 126 were characterized as abundant taxa, with relative abundances over 0.1%. Proteobacteria, mainly Alphaproteobacteria, Betaproteobacteria, and Gammaproteobacteria dominated all samples collected. Bacteroidetes were in high abundance in Antarctic island samples but virtually absent from continental Antarctica samples. On the other hand, high abundances of Firmicutes were recorded in continental Antarctica (Figure 4A,B). Actinobacteria decreased in abundance at SkyBlu and Pine Island Bay but not in the Ellsworth samples. Bacteroidetes changed from Cytophaga, Flavobacteria, Sphingobacteriia, and Chitinophagia in northern sites, through unidentified at SkyBlu and Pine Island Bay, to Chitinophaga only at the southern sites.

Overall, at the genus level, distinctions between each site were clear (Supplementary Figure S1). A few instances of notable variations included the high proportion of *Methylobacterium* and *Rhizobium* in continental Antarctica (up to 20 and 13%, respectively), which are present in very low abundances in the Antarctic islands.

The core microbiome, as comprised of OTUs identified in over 80% of all samples analyzed, was represented by only one OTU, classified as *Bradyrhizobiaceae*, at a total relative abundance of 0.52%. The indicator species analysis identified OTUs specifically associated with geographic locations. Although over six thousand OTUs were detected in total, only 38 were associated with Signy Island, mainly in the Alphaproteobacteria, Actinobacteria and Bacteroidetes. 48 indicator species OTUs were identified among Livingston Island samples, mainly Betaproteobacteria, Bacteroidetes and Alphaproteobacteria. No indicator species were identified for continental Antarctic sites, further highlighting the difference in microbial communities between each location, regardless of geographic distances.

### Cell Viability and Impact of Relic DNA on Snow Microbiological Diversity

**FIGURE 5:**
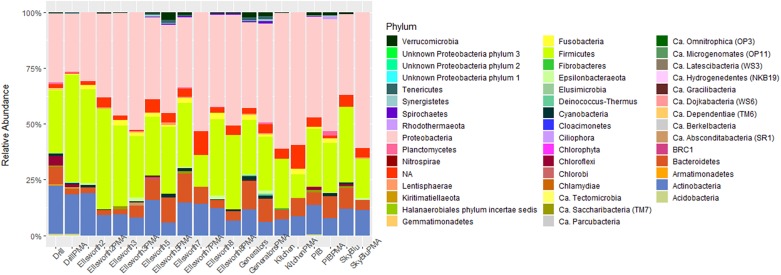
Bar chart of microbial diversity at the phylum level between PMA and non-PMA-treated samples.

To investigate the impact of relic DNA from dead cells, on microbial diversity of Antarctic snow, the SkyBlu, Pine Island Bay, Ellsworth pristine, camp and drill samples were treated with PMA before DNA extraction and sequencing. The number of OTUs, Shannon index and Simpson index were all lower in PMA-treated samples than in non-PMA-treated samples (Supplementary Figure S2), showing the presence of relic DNA in snow. While the number of OTUs identified decreased by up to 50% after PMA treatment in some cases (Supplementary Figure S2A), differences in diversity indices by treatment were not significant (ANOVA *F*_1,18_ = 1.1, *p* > 0.05) (Supplementary Figures S2B,C). Similarly, the community composition did not significantly change after PMA treatment (ANOSIM, *R* = 0.06, *p* = 0.125). Differences in diversity were mainly characterized by the decrease in Actinobacteria, an increase in Proteobacteria and variation in Firmicute abundance in PMA-treated samples (Figure 5).

### The Lake Ellsworth Site

For this site, the Antarctic snow community was determined from an area of current scientific research interest. We sampled surface snow within the Lake Ellsworth deep-field camp in December 2012 (Pearce D.A. et al., 2016), close to the kitchen, generators and the deep hot-water drilling head, and away from the camp to use as a proxy for pristine snow communities. This deep field site was characterized by human presence, activity and disturbance of the natural environment. This is reflected by the isolation of the drilling head site in the PCoA (Figure 2A and Supplementary Figure 3), with an unique microbial assemblage. While both off-site and camp samples were highly similar in richness and diversity, the drilling site harbored a higher number of OTUs and appeared much more diverse than any other site sampled, including Signy and Livingston Islands (Figure 2). Differences in the microbial communities of the drilling site were apparent at the genus level (Supplementary Figure 1), and this is further illustrated by the indicator species analysis, which identified 49 drill-associated taxa, mainly classified as Firmicutes, Actinobacteria and Alphaproteobacteria. A BLAST search identified many of these taxa as soil-related, often with 99% identity match to worldwide soils, from Brazil to the Chinese mountains of Yanshan, to Moab National Park and Mt. Kilimanjaro.

## Discussion

Our study of microbial communities in Antarctic snow identified three clusters: Livingston Island, Signy Island and continental Antarctica, which included Pine Island Bay, SkyBlu and the Lake Ellsworth region (Figure 3). The differences likely result from geographical distance and variation in environmental conditions from the Antarctic islands to continental Antarctica. Livingston Island has an annual mean temperature of −1°C by the coast and is characterized by seasonal snow cover and low altitude (Hodson et al., 2017). Similarly, Signy Island has mean annual temperatures around −3.5°C also with seasonal snow cover (Guglielmin et al., 2008). The sampling locations around continental Antarctica are at high altitude, with annual mean temperatures between −20 and −30°C (Bohlander and Scambos, 2001) and permanent snow cover. Therefore, the difference in snow microbial communities between the Antarctic islands and continental Antarctica are likely due to snow type, density and physicochemical properties (Bertler et al., 2005; Masson-Delmotte et al., 2008). Differences observed between sites within the same cluster reflect natural variation of microbial communities, which have been observed down to the pore scale in soils (Ruamps et al., 2011).

Overall, Alphaproteobacteria, Betaproteobacteria, and Gammaproteobacteria were abundant in all samples, which is in accordance with previous studies on the Antarctic snow microbial community (Michaud et al., 2014; Antony et al., 2016; Lopatina et al., 2016). However, compared to previous studies, Firmicutes were consistently identified in all samples and Cyanobacteria were generally below the 0.1% abundance threshold. Firmicutes are known to harbor a number of survival strategies such as biofilm formation or the formation of endospores. These strategies allow them to survive extreme environmental conditions such as physicochemical gradients, UV radiation and desiccation (Filippidou et al., 2016).

In all samples, a large proportion of microorganisms were identified as soil-related. The analysis of extraction kit controls suggested that they were unlikely to be the result of kit contamination, as all samples, except SkyBlu and Blank 2, had more than 2000 reads. Beside SkyBlu and Blank2, PMA-treated samples and Blank1 had the smallest number of reads (below 10,000 reads). Furthermore, the blanks clustered away from other samples in the PCoA, illustrating the differences in microbial communities (Supplementary Figure 3). All other samples had over 12,000 reads; above the 2,000 minimum reads required to attain reproducible and reliable patterns of diversity (Caporaso et al., 2010b).

The study by Cameron et al. (2015) identified soil microorganisms as a primary source of the snow microbial assemblage of the West Greenland Ice Sheet, while studies on airborne microbial communities have reported soil ecosystems as a primary source of microorganisms in the Arctic (Cuthbertson et al., 2017; Šantl-Temkiv et al., 2018). Airborne microbial communities are consistently dominated by Firmicutes, Alpha-, Beta-, and Gamma- Proteobacteria (Fierer et al., 2008) including in Antarctica (Pearce et al., 2010; Bottos et al., 2014). Spore-forming genera (i.e., *Bacillus* spp.) are a major component of airborne microbial communities and are associated with long-distance dispersal (Kellogg et al., 2004). Our study shows that snow and airborne microbial communities appear similar, especially in continental Antarctica, including a high abundance of Bacilli (Figure 4B) suggesting the importance of long-distance dispersal in seeding continental Antarctic snow ecosystems. However, we identified a small abundance of Bacteroidetes in the Ellsworth snow samples. This could reflect small terrestrial inputs from the Ellsworth Mountains, dominated by Bacteroidetes (Yergeau et al., 2007) and the closest terrestrial system, located approximately 70 km away from the sampling site (Pearce D.A. et al., 2016).

Antarctic island snow communities had higher abundances of Bacteroidetes and Actinobacteria and lower abundances of Firmicutes. Antarctic soil microbial communities from both Signy and Livingston Islands, harbored high abundances of Bacteroidetes, including Sphingobacteria and Chitinophagia (Yergeau et al., 2007; Chong et al., 2009, 2010; Ganzert et al., 2014), also identified in the collected snow samples (Figure 4B). This may reflect local dispersal mechanisms, such as vector-based dispersal through the local wildlife (Green and Bohannan, 2006), or aerosolization of local soil bacteria dispersed over short-distances (Martiny et al., 2006). It should be noted that soil systems occur on Antarctic islands and the snow cover is seasonal, while in continental Antarctica, snow cover is permanent, soil ecosystems are sparse and, often, the closest source is the coastline or the Antarctic islands themselves. Overall, the observed microbial communities and clustering pattern support the hypothesis that soils are the primary source of snow microorganisms through local or long-range dispersal. While Antarctic islands snow microbial communities may be influenced by both pathways (Vincent, 2000), continental Antarctica appears to be seeded by air-borne microorganisms through long-range aeolian transport.

The indicator species analysis identified limited OTUs with geographic associations. The majority of indicator OTUs were associated with the Antarctic islands and far fewer with continental Antarctica. This analysis suggests that Signy and Livingston Islands have unique, geographically associated communities, but locally homogeneous microbial communities, likely influenced by surrounding environmental conditions. Continental sites with harsher environmental conditions, primarily seeded by air-borne microorganisms, had more variable microbial communities, even within the same location, likely dependant on weather and wind patterns.

We assessed the presence of relic DNA in snow samples collected from continental Antarctica. Relic DNA coming from dead cells, has been identified representing on average 40% of DNA extracted from soil samples (Carini et al., 2017; Lennon et al., 2018). While studies of microbial communities are essential to understand functional activities and ecological roles of microorganisms, relic DNA could lead to false estimations of putative functions and lead to misunderstanding of the role of microorganisms in geochemical cycling. Identifying relic DNA in environmental samples is relatively new and focused on soils (Carini et al., 2017; Fierer, 2017), where microbial biomass is high and concentrated. As samples were filtered prior to PMA treatment and extraction, free environmental DNA would have been discarded during the filtering process, unless attached to particles and, as such, relic DNA would only be present in low abundance in snow samples. Yet, in some cases, the number of reads and identified OTUs was reduced by 50% while, in others, differences were imperceptible. Despite variation in OTUs between non-PMA and PMA-treated samples, alpha diversity and community composition did not change significantly. While relic DNA can deeply affect some microbial community analyses, relic DNA is not a major issue in snow samples.

Finally, we did not identify directly human-related taxa in any of the samples. This is likely due to environmental conditions preventing human bacteria from colonizing Antarctic snow ecosystems rather than the lack of contamination of the surrounding environment (Cowan et al., 2011). However, strong winds and snow movements could have potentially transported human-related bacteria to distant sites and, thus, would remain undetected in this study. Furthermore, we used sites from distant locations as a proxy for the initial indigenous microbial populations present before human disturbance at the Ellsworth camp site. The high similarity of microbial assemblages in the Ellsworth off-site samples and camp samples suggests that human presence may not have a major impact on snow microbial communities. However, strong human-derived disturbances and activities, such as the import of equipment, snow digging, intense stepping, and sitting and manipulating instruments, may lead to changes in natural assemblages, as illustrated at the drilling site. While it is unlikely that the drilling site communities differ from the other samples purely by chance, we cannot exclude it completely as we did not sample the site prior to the research activity. In addition to human activities impacting the indigenous snow microbial community, the presence of exogenous microorganisms on camp and drilling material could also have an impact. This could be mitigated in future by careful and thorough cleaning and sterilization of equipment prior to landing in Antarctica and throughout the drilling process (Priscu et al., 2013; Makinson et al., 2016). Due to the observed differences in microbial communities from site to site, we recommend indigenous surface microbial communities be considered in planning deep-field subsurface access operations and characterized prior to research activities.

## Conclusion

This study, of spatial variation in microbial communities from Signy Island down toward the Ellsworth Mountains, covers the largest geographical extent of any investigation of Antarctic snow microbial communities. We identified three distinct geographic regions from the analysis, with each Antarctic island as individual clusters, and all sites of continental Antarctica clustered together. While each cluster harbored distinct microbial assemblages, within-cluster differences were also observed, illustrating the spatial variability of Antarctic snow bacterial communities. Overall, communities were primarily composed of soil-related microorganisms, suggesting colonization from both local and distant terrestrial ecosystems. The Lake Ellsworth case study suggests that while low-level human presence may not affect natural systems, intense human activities and ecosystem disturbances might. When undertaking research aimed at detecting life in extreme environments, such as in subglacial lakes, we believe it is important to consider how the indigenous microbial communities may be affected across the field site as well as with in subglacial targets themselves (Siegert and Kennicutt, 2018).

## Author Contributions

LM conducted the bioinformatics processing, statistical analyses, and wrote the manuscript. MS conducted the fieldwork, laboratory work of the Signy samples, and contributed to bioinformatic processing. IM and MM conducted the laboratory work from the Ellsworth, Pine Island Bay, and SkyBlu samples. MT participated in the Ellsworth sampling campaign. MJS was the Principal Investigator of the Lake Ellsworth exploration program. DP designed the study, co-wrote the manuscript and co-ordinated the sampling campaign. All authors have read, revised, and approved the manuscript.

## Conflict of Interest Statement

The authors declare that the research was conducted in the absence of any commercial or financial relationships that could be construed as a potential conflict of interest.

## Supplementary Material

FIGURE S1 Stacked bar chart of the bacterial diversity by sampling site.

FIGURE S2 Plots of Alpha diversity measures by Location and by Treatment. **(A)** Observed OTUs. **(B)** Shannon Index. **(C)** Simpson Index.

FIGURE S3 PCoA of microbial communities focused on the continental Antarctica cluster. It includes samples from the Ellsworth Camp, drilling site and extraction blanks clustered away.

TABLE S1 Metadata table of sample sites and GPS locations.
