# Supplementary figures and images for: Spatial Variability of Antarctic Surface Snow Bacterial Communities
Source: Front Microbiol. 2019 Mar 26;10:461. doi: 10.3389/fmicb.2019.00461 (PMC6443967; doi:10.3389/fmicb.2019.00461)

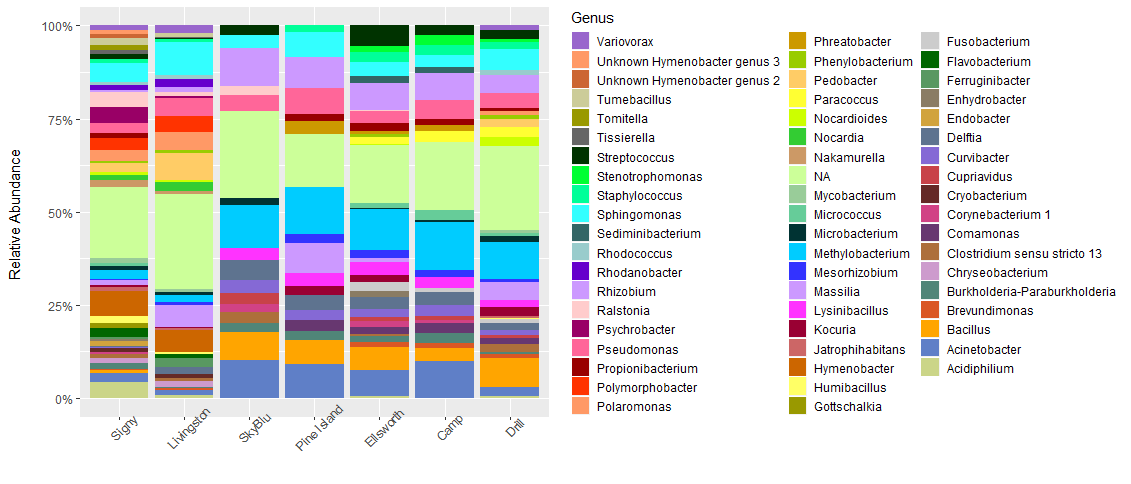

Supplement: FIGURE S1 — Stacked bar chart of the bacterial diversity by sampling site. [file Image_1.TIFF]

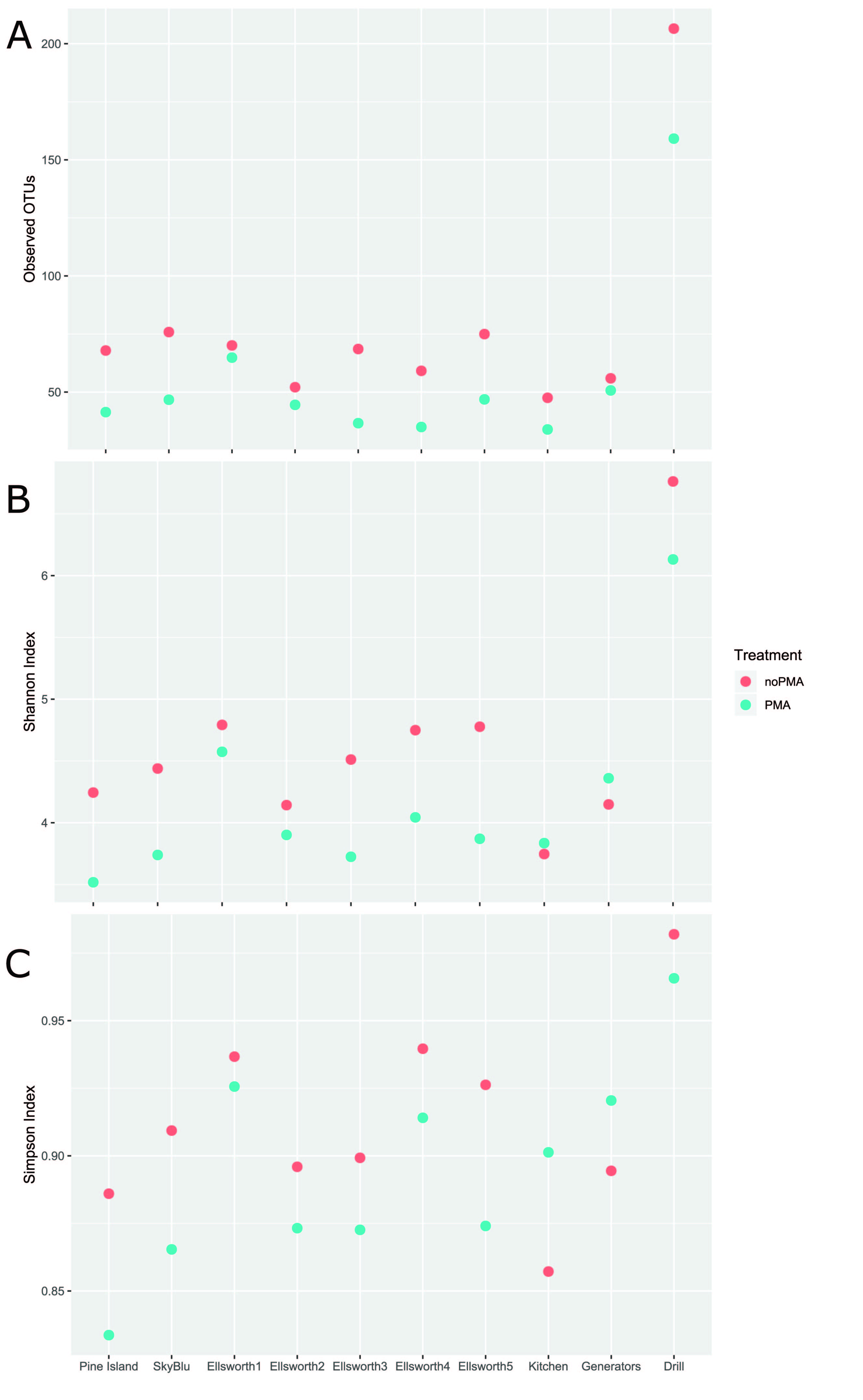

Supplement: FIGURE S2 — Plots of Alpha diversity measures by Location and by Treatment. (A) Observed OTUs. (B) Shannon Index. (C) Simpson Index. [file Image_2.JPEG]

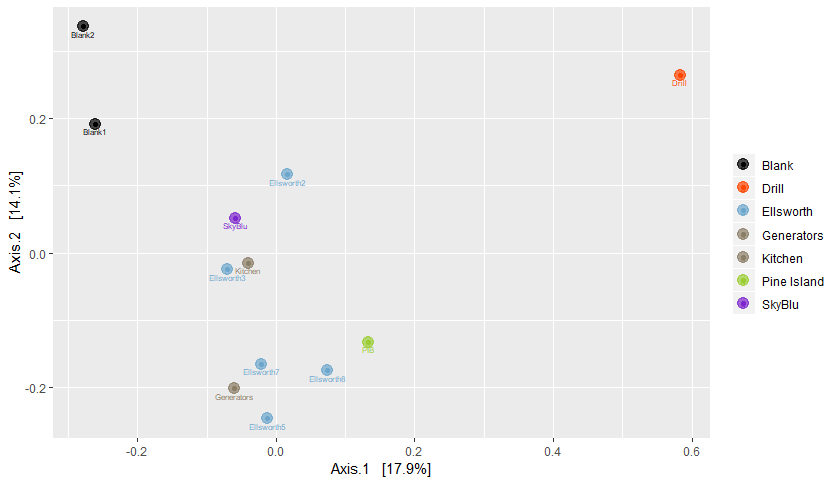

Supplement: FIGURE S3 — PCoA of microbial communities focused on the continental Antarctica cluster. It includes samples from the Ellsworth Camp, drilling site and extraction blanks clustered away. [file Image_3.TIFF]
